# The anti-inflammatory and antioxidant activity of 25 plant species used traditionally to treat pain in southern African

**DOI:** 10.1186/s12906-015-0669-5

**Published:** 2015-05-27

**Authors:** Salmon A. Adebayo, Jean P. Dzoyem, Leshweni J. Shai, Jacobus N. Eloff

**Affiliations:** Phytomedicine Programme, Department of Paraclinical Sciences, Faculty of Veterinary Science, University of Pretoria, Private Bag X04, Onderstepoort, Pretoria, 0110 South Africa; Dept of Biomedical Sciences, Faculty of Science, Tshwane University of Technology, Private Bag X680, Pretoria, 0001 South Africa; Department of Biochemistry, Faculty of Science, University of Dschang, P.O. Box 67, Dschang, Cameroon

**Keywords:** *Anti-inflammatory*, *Medicinal plants*, *15-lipoxygenase*, *Nitric oxide*, *Peltophorum africanum*

## Abstract

**Background:**

Inflammation is a common risk factor in the pathogenesis of conditions such as infections, arthritis, type 2 diabetes mellitus, obesity and cancer. An ethnobotanical survey of medicinal plants used traditionally to treat inflammation and related disorders such as pain, arthritis and stomach aches in southern Africa led to the selection of 25 plant species used in this study.

**Methods:**

The antioxidant activities of acetone extracts were determined by measuring the free radical scavenging activity and ferric reducing ability, respectively. The anti-inflammatory activities of the extracts were determined by measuring the inhibitory effect of the extracts on the activities of the pro-inflammatory enzyme, lipoxygenase and inducible nitric oxide synthase.

**Results:**

Extracts of *Peltophorum africanum* had good antioxidant activity with IC_50_ values of 4.67 ± 0.31 μg/mL and 7.71 ± 0.36 μg/mL compared to that of the positive control ascorbic acid (2.92 ± 0.14 μg/mL and 13.57 ± 0.44 μg/mL), using the 2,2-diphenyl-1-picrylhydrazyl (DPPH) scavenging and 2,2′-azinobis (3-ethylbenzthiazoline-6-sulphonic acid (ABTS) methods, respectively. The metabolism of linoleic acid to leukotriene derivatives by 15-lipoxygenase (15-LOX) was also inhibited by the crude acetone extracts of *Peltophorum africanum* (IC_50_ = 12.42 μg/mL), *Zanthoxylum capense* (IC_50_ = 14.92 μg/mL) compared to the positive control quercetin (IC_50_ = 8.75 μg/mL). There was a poor correlation between the flavonoid content and 15-LOX inhibition by the extracts (R^2^ = 0.05), indicating that flavonoids are not involved in LOX inhibition. Extracts of *Clausena anisata,* at a concentration of 6.25 μg/mL inhibited nitric oxide production by RAW 264.7 macrophage cell lines in vitro by 96 %. The extracts of *Zanthoxylum capense* were the least cytotoxic (IC_50_ > 1000 μg/mL) when the extract toxicity was determined against Vero (African green Monkey) kidney cell lines.

**Conclusion:**

Some plant species used traditionally to treat pain have reasonable anti-inflammatory activity and flavonoids are probably not involved in this process.

## Background

Medicinal plants have long been recognised as important sources of therapeutically active compounds. Evidence-based research supports the medical and pharmacological benefits of plant-derived compounds, with increasing interest in the identification and characterization of bioactive compounds from natural sources [1].

One of the earliest recorded approaches for treating inflammation and pain was the application of extracts from willow leaves by Celsius in 30 AD [2]. This observation led to the discovery of acetyl salicylic acid, the active component of aspirin, a major anti-inflammatory drug widely used in clinical practice, along with many other non-steroidal anti-inflammatory drugs (NSAIDs) in current use [3].

Non-steroidal anti-inflammatory drugs are commonly prescribed for treatment of pain and inflammatory conditions such as rheumatoid arthritis, osteoporosis and Alzheimer’s disease. However, because many NSAIDs are associated with side effects such as gastrointestinal bleeding and suppressed function of the immune system [4], attention has shifted to alternative pharmacotherapies [5, 6]. Recent studies on *Zingiber officinale*, ginger, suggest that it might be as effective as some NSAIDs in the treatment of inflammation and related pain [7, 8].

In South Africa the use of plants to treat many diseases is widely practiced. According to Iwalewa et al. [9], more than 115 plant species of 60 families are used in South Africa to treat pain-related inflammatory disorders in humans and animals. The bioactive principles in these plant species have been linked to secondary metabolites such as phenolic compounds (curcumins, flavonoids and tannins), saponins, terpenoids and alkaloids [9, 10,]. Biological and therapeutic properties attributed to these plant metabolites include antioxidant, anti-inflammatory, antimicrobial and anticancer activities [10]. The mechanisms of action of many phenolic compounds such as flavonoids, tannins and curcumins are thought to be via their free radical scavenging activities or the inhibition of pro-inflammatory enzymes such as cyclo-oxygenases (COX) and lipoxygenases (LOX) in the inflammatory cascades [11, 12].

Flavonoids are a group of polyphenols thought to inhibit the biosynthesis of prostaglandins, end-products in the COX and LOX pathways of immunologic responses [13]. There are three known isomeric-forms of COX i.e. COX-1 and COX-2, with a recently described third isomeric-form, COX-3 that is selectively inhibited by acetaminophen and related compounds [14, 15]. The selective inhibition of COX-2 is more desirable because the inhibition of COX-1 in the gastric mucosa is associated with the undesirable effects of NSAIDs [16]. COX-2 is induced as an early response to pro-inflammatory mediators and stimuli such as endotoxins and cytokines [17]. Upon induction, COX-2 synthesizes prostaglandins that contribute to inflammation, swelling and pain [18]. Consequently, dual COX-2/LOX inhibitor compounds could potentially be developed into safer and more effective drugs for the treatment of inflammation since they could potentially inhibit biosynthesis of prostaglandins and leukotrienes respectively from arachidonic acid [16, 19], without the undesirable effects of NSAIDs.

Lipoxygenases are lipid-peroxidizing enzymes involved in the biosynthesis of leukotriene from arachidonic acid, mediators of inflammatory and allergic reactions. These enzymes catalyse the addition of molecular oxygen to unsaturated fatty acids such as linoleic and arachidonic acids [20]. There are four main iso-enzymes already described, namely, 5-LOX, 8-LOX, 12-LOX and 15-LOX, depending on the site of oxidation in the unsaturated fatty acids [20]. The common substrates for LOX are linoleic and arachidonic acids. For many in vitro studies, soy bean LOX is used due to difficulties in obtaining human LOX for bioassays [21].

During inflammation, arachidonic acid is metabolized via the COX pathway to produce prostaglandins and thromboxane A_2_, or via the LOX pathway to produce hydroperoxy-eicosatetraenoic acids and leukotrienes [22]. The LOX pathway is active in leucocytes and many immune-competent cells including mast cells, neutrophils, eosinophils, monocytes and basophils. Upon cell activation, arachidonic acid is cleaved from cell membrane phospholipids by phospholipase A_2_ and donated by LOX activating protein to LOX, which then metabolises arachidonic acids in a series of reactions to leukotrienes, a group of inflammatory mediators [23]. Leukotrienes act as phagocyte chemo-attractant, recruiting cells of the innate immune system to sites of inflammation. For instance in an asthmatic attack, it is the production of leukotrienes by LOX that causes the constriction of bronchioles leading to bronchospasm [8, 16]. Therefore, the selective inhibition of LOX is an important therapeutic strategy for asthma [8, 16, 24]. Inhibitors of the activities of LOX could provide potential therapies to manage many inflammatory and allergic responses. Medicinal plants may therefore be potential sources of inhibitors of COX-2/LOX that may have fewer side effects than NSAIDs [24].

Nitric oxide (NO) is a short-lived free radical that mediates many biological processes. One of the functions of NO is to enhance the bactericidal and tumoricidal activities of activated macrophages [25, 26]. Excessive production of NO could however potentially lead to tissue damage and activation of pro-inflammatory mediators [27, 28]. The potential of extracts from medicinal plants to scavenge these free radicals and modulate inflammatory reactions has been demonstrated [29–31].

The objective of this study was to determine the anti-inflammatory activity of extracts in relevant bioassays in order to validate their use for pain relief and to identify plants that could be investigated in more detail.

## Methods

Analytical grade chemicals were purchased from various suppliers in South Africa, and were used for the bioassays in the laboratory.

### Preparation of plant materials

Fresh leaves of the selected plants species were collected from the Manie van der Schijff Botanical Garden, University of Pretoria in March 2012. The plant materials were dried at room temperature in a well-ventilated room for a week. After drying, the materials were ground to fine powder using a MacSalab Model 200 grinder and stored in closed honey jars in the dark. Herbarium specimens for each of the plant species were prepared and deposited at HGWJ Schweickerdt Herbarium, University of Pretoria. Herbarium voucher specimen numbers (PRU voucher numbers) are provided in Table 1.

**Table 1:** Percentage crude extract yield from the selected plant species

Plant species	Family name	Herbarium specimen no.	Common names	Medicinal uses	Yield (mg)	Yield (%)
*Acacia burkei*	Fabaceae	PRU/120581/1/Adebayo SA	Black monkey thorn	Painful back and eye [10]	110	3.7
*Acacia sieberiana*	Fabaceae	PRU/120582/1/Adebayo SA	Paperback thorn	Fever, back aches and pain [10]	210	7.0
*Acokanthera oppositifolia*	Apocynaceae	PRU/120583/1/Adebayo SA	Bushman’s poison	Headaches and pain [56]	520	17.3
*Bridelia micrantha*	Phyllanthaceae	PRU/120584/1/Adebayo SA	Coast gold leaf	Anti-inflammatory, abdominal pain [57]	340	11.3
*Clausena anisata*	Rutaceae	PRU/120585/1/Adebayo SA	Maggot-killer	Abdominal pain, fever, rheumatism [58]	190	6.3
*Dichrostachys cinerea*	Fabaceae	PRU/120586/1/Adebayo SA	Sickle bush	Analgesic [59, 60]	50	1.7
*Ekebergia capensis*	Meliaceae	PRU/120587/1/Adebayo SA	Cape ash	Headaches, backaches and cough [58]	120	4.0
*Erythrophleum lasianthum*	Fabaceae	PRU/120588/1/Adebayo SA	Thornless tree	Headaches [59]	350	11.6
*Harpephyllum caffrum*	Anacardiaceae	PRU/120589/1/Adebayo SA	Wild plum or bush mango	Pain alleviation [60]	120	4.0
*Kigelia africana*	Bignoniaceae	PRU/120590/1/Adebayo SA	Sausage tree	Analgesics, fever rheumatism [61]	60	2.0
*Melianthus comosus*	Melianthaceae	PRU/120591/1/Adebayo SA	Honey flower	Rheumatism [59]	260	8.7
*Peltophorum africanum*	Fabaceae	PRU/120592/1/Adebayo SA	African/weeping wattle	Chronic pains [62]	100	3.3
*Pittosporum viridiflorum*	Pittosporaceae	PRU/120593/1/Adebayo SA	Cheesewood	Abdominal pains [58]	140	4.6
*Plumbago auriculata*	Plumbaginaceae	PRU/120594/1/Adebayo SA	Plumbago	Headaches and malaria relief [58]	80	2.7
*Polygala fruticosa*	Polygalaceae	PRU/120595/1/Adebayo SA	Butterfly bush	Venereal diseases [60]	570	19.0
*Ptaeroxylon obliquum*	Ptaeroxylaceae	PRU/120596/1/Adebayo SA	Sneezewood	Headaches [60]	200	6.7
*Rhus chirindensis*	Anacardiaceae	PRU/120597/1/Adebayo SA	Red currant	Arthritis, pain [62]	200	6.7
*Sclerocarya birrea*	Anacardiaceae	PRU/120598/1/Adebayo SA	Marula	Anti-inflammatory, fever [62]	150	5.0
*Tecomaria capensis*	Bignoniaceae	PRU/120599/1/Adebayo SA	Cape honey suckle	Abdominal pains [59]	170	5.7
*Terminalia phanerophlebia*	Combretaceae	PRU/120600/1/Adebayo SA	Lebombo cluster leaf	Aches, wounds and infections [41, 63]	210	7.0
*Trichilia dregeana*	Meliaceae	PRU/120601/1/Adebayo SA	Thunder tree	Stomach ailment and backaches [58]	70	2.3
*Terminalia prunioides*	Combretaceae	PRU/120602/1/Adebayo SA	Lowveld cluster leaf	Abdominal pains, backaches [41]	170	5.7
*Tulbaghia violacea*	Alliaceae	PRU/120603/1/Adebayo SA	Wild garlic	Pain relief and fever [64]	660	22.0
*Warburgia salutaris*	Canellaceae	PRU/120604/1/Adebayo SA	Pepper-bark tree	Headaches, influenza and fever [65]	150	5.0
*Zanthoxylum capense*	Rutaceae	PRU/120605/1/Adebayo SA	Small knobwood	Abdominal pains [65]	220	7.3

### Preparation of crude extracts for biological assays

Ground leaf powders (3 g) were extracted in 30 mL of 70 % acetone in clean honey jars and vigorously shaken for 3 h (Labotec model 20.2 shaker). The crude acetone extracts were filtered through Whatman No. 1 filter papers into pre-weighed honey jars, and then left open overnight for solvent evaporation. The honey jars were weighed again to determine the percentage yield of the crude extracts. For the biological assays, the crude extracts were reconstituted in dimethyl sulphoxide (DMSO) at a concentration of 10 mg/mL.

### Determination of total phenolics and flavonoids

Total phenolics were determined according to the method of Folin-Ciocalteu described by Makkar [32], with slight amendments. In brief, 25 μL of crude extract was treated with 250 μL Folin-Ciocalteu reagent for 5 min. The reaction was stopped by adding 750 μL 20 % anhydrous sodium carbonate. The volume was made up to 5 mL with distilled water and incubated in the dark at room temperature for 2 h. After incubation, the absorbance was read at 760 nm with a spectrophotometer (HELIOS βT60, Separation Scientific). The phenolic content was determined from a standard curve of different concentrations of gallic acid DMSO. The results were expressed as mg/g gallic acid equivalent (GAE).

Flavonoid content of the extracts was determined using the methods of Yadav and Agarwala, [33], also amended slightly. Crude extracts (100 μL) were dissolved in 300 μL methanol, to which 20 μL 10 % aluminium chloride was added. A further 20 μL of 1 M sodium acetate was added to the solution. The resultant solution was made up to 1 mL with distilled water. This was incubated at room temperature for 30 min in a microplate. After incubation, the absorbance was read at 450 nm in a microplate reader (SpectraMax 190, Molecular devices). Quercetin (10 mM) was used as a standard. The flavonoid content of each extract was expressed as mg/g quercetin equivalent (QE).

### The 2, 2-Diphenyl-1-picrylhydrazyl (DPPH) radical scavenging assay methods

The DPPH radical-scavenging activity was determined using the method of Brand-Williams et al. [34]. Ascorbic acid and Trolox were used as positive controls, methanol as negative control and extract without DPPH as blank. Results were expressed as percentage reduction of the initial DPPH absorption in relation to the control. The concentration of extract leading to 50 % reduction of DPPH (IC_50_) was also determined.

### The 2, 2′-azinobis (3-ethylbenzthiazoline-6-sulphonic acid) (ABTS) radical scavenging assay methods

The ABTS radical scavenging capacity of the samples was measured with modifications of the 96-well microtitre plate method described by Re et al. [35]. Trolox and ascorbic acid were used as positive controls, methanol as negative control and extract without ABTS as blank. The percentage of ABTS• + inhibition was calculated using the formula:

$$ \mathrm{Scavenging}\ \mathrm{capacity}\ \left(\%\right)=100-\left[\frac{{\mathrm{OD}}_{\mathrm{sample}}\hbox{-} {\mathrm{OD}}_{\mathrm{sample}\ \mathrm{blank}}}{{\mathrm{OD}}_{\mathrm{control}}\hbox{-} {\mathrm{OD}}_{\mathrm{control}\ \mathrm{blank}}}\right]\times 100\% $$

where OD represents the optical density or absorbance.

The IC_50_ values were calculated from the graph plotted as inhibition percentage against the concentration.

### The ferric reducing ability of plasma (FRAP) assay methods

The FRAP assay was carried out according to the procedure of Benzie and Strain [36] with slight modification. The FRAP assay depends upon the reduction of ferric tripyridyltriazine (Fe (III)-TPTZ) reduction to ferrous tripyridyltriazine (Fe (II)-TPTZ) by a reductant at low pH. Ferrous (II)-TPTZ has an intensive blue colour and can be monitored at 593 nm. Briefly, the FRAP reagent was prepared using an acetate buffer (pH 3.6), 10 mM TPTZ solution in 40 mM hydrochloric acid and 20 mM iron (III) chloride solution in proportions of 10:1:1 (v/v), respectively. Twenty five microliters of sample were added to 175 μL of the FRAP reagent. The absorbance of the reaction mixture was recorded at 593 nm (SpectraMax 190, Molecular devices) after 5 min. The standard curve was made using iron (II) sulphate solution (40–0.078 μg/mL), and the results were expressed as μg Fe (II)/g of extract. All the measurements were taken in triplicate and the mean values were calculated.

### Inhibition of 15-lipoxygenase (15-LOX) enzyme

The 15-LOX (Sigma) was made up to a working solution of 200 units/mL and kept on ice. A volume of 12.5 μL of test sample or control (dissolved in DMSO) was added to 487.5 μL of 15-LOX in a 96-well microtitre plate and incubated at room temperature for 5 min. After incubation, 500 μL substrate solutions (10 μL linoleic acid dissolved in 30 μL ethanol, made up to 120 mL with 2 M borate buffer at pH 9.0) was added to the solution. After 5 min incubation at room temperature, the absorbance was measured with the microplate reader at 234 nm (SpectraMax 190, Molecular devices). Quercetin (1 mg/mL) was used as a positive control, while DMSO was used as the negative control (100 % enzyme activity or no enzyme inhibition). The percentage enzyme inhibition of each extract compared with negative control as 100 % enzyme activity was calculated using the equation;

$$ \%\ \mathrm{Inhibition}=\frac{\left({\mathrm{OD}}_{\mathrm{extract}}\hbox{-} {\mathrm{OD}}_{\mathrm{blank}}\right)}{\left({\mathrm{OD}}_{\mathrm{negative}\ \mathrm{control}}\hbox{-} {\mathrm{OD}}_{\mathrm{blank}}\right)}\times 100\% $$

The results were expressed as IC_50,_ i.e. concentration of the extracts and controls that resulted in 50 % 15-LOX inhibition plotted on a graph.

### Inhibition of nitric oxide (NO) production

#### Cell culture

The RAW 264.7 macrophage cell lines obtained from the American Type Culture Collection (Rockville, MD, USA) were cultured in plastic culture flasks in Dulbecco’s Modified Eagle’s Medium (DMEM) containing l-glutamine supplemented with 10 % foetal calf serum (FCS) and 1 % PSF (penicillin/streptomycin/fungizone) solution under 5 % CO_2_ at 37 °C, and were split twice a week. Cells were seeded in 96 well-microtitre plates and were activated by incubation in medium containing LPS (5 μg/mL) and various concentrations of extracts dissolved DMSO.

### Measurement of nitrite

Nitric oxide released from macrophages was assessed by the determination of nitrite concentration in culture supernatant using the Griess reagent. After 24 h incubation, 100 μL of supernatant from each well of cell culture plates was transferred into 96-well microtitre plates and equal volume of Griess reagent was added. The absorbance of the resultant solutions in the wells of the microtitre plate was determined with a microtitre plate reader (SpectaMax 190 Molecular devices) after 10 min at 550 nm. The concentrations of nitrite were calculated from regression analysis using serial dilutions of sodium nitrite as a standard. Percentage inhibition was calculated based on the ability of extracts to inhibit nitric oxide formation by cells compared with the control (cells in media without extracts containing triggering agents and DMSO), which was considered as 0 % inhibition.

### Cell viability

To ensure that the observed nitric oxide inhibition was not due to cytotoxic effects, the cytotoxicity was also determined against Vero Monkey kidney cells as previously described by Mosmann [37], with slight modifications. After removal of media, the cells were topped up with 200 μL DMEM. To each well, 30 μL of 15 mg/mL 3-(4, 5-dimethylthiazol-2-yl)-2, 5-diphenyl tetra-zoliumbromide (MTT) was added. The cells were incubated at 37 °C with 5 % CO_2_. After 2 h, the medium was carefully discarded and the formed formazan salt was dissolved in DMSO. The absorbance was read at 570 nm (SpectraMax 190, Molecular devices). The percentage of cell viability was calculated with reference to the control (cells without extracts containing LPS taken as 100 % viability).

All the experiments to measure nitric oxide inhibition were conducted three times in triplicate.

### Cytotoxicity assessments

The cytotoxicity of the extracts (dissolved in acetone) against Vero monkey kidney cells was assessed by the MTT reduction assay as previously described [37] with slight modifications. Cells were seeded at a density of 1 × 10^5^ cells/mL (100 μL) in 96-well microtitre plates and incubated at 37 °C and 5 % CO_2_ in a humidified environment. After 24 h incubation, extracts (100 μL) at varying final concentrations were added to the wells containing cells. Doxorubicin (40–0.38 μM) was used as a reference compound. A suitable blank control with equivalent volume of acetone was also included and the plates were further incubated at 37 °C for 48 h in a CO_2_ incubator. The medium was removed by aspiration and cells were then washed twice with PBS, followed by suspension in fresh medium (200 μL). Then, 30 μL of MTT (5 mg/mL in PBS) was added to each well and the plates were incubated at 37 °C for 4 h. The medium was removed by aspiration and 100 % DMSO (100 μL) added to dissolve the formed formazan crystals. The absorbance was measured on SpectraMax 190 (Molecular devices) microtitre plate reader at 570 nm. The percentage of cell growth inhibition was calculated based on a comparison with untreated cell. The selectivity index (SI) values were calculated by dividing cytotoxicity LC_50_ values by the MIC values (SI = LC_50_/MIC).

### Statistical analysis

All results are presented as the means of triplicate experiments. Differences between test extracts in these experiments was assessed for significance using analysis of variance (ANOVA) and student *t*-test, where probability (p ≤ 0.05) was considered significant.

## Results and discussion

The results obtained in this study are presented below using Tables and Figures for ease of interpretation and data comparison.

### Crude yield of extracts

*Tulbaghia violacea* yielded 22 % of crude acetone extract from 3 g plant material, the highest yield of all the plant species in this study. This plant grows as a bulbous rhizome, which had to be cut into pieces for proper drying. The presence of reserve materials might account for the high yield of extract from the plant unlike the other plant species in the study, whose leaves could be easily dried when left open in the drying room for three days (Table 1).

### Total phenolics and flavonoid contents

The high extract yield from *T. violacea* did not correlate well with its total phenolics and flavonoid content. This may be due to high concentrations of carbohydrates as reserve material in the rhizome. *Terminalia phanerophlebia* and *Terminalia prunioides* with lower crude extract yield of 7 % and 5.7 % respectively contained more total phenolics than *T. violacea* (Table 1). The highest amounts of total phenolic compounds were obtained from *T. phanerophlebia* (86 mg/g GAE) followed by *T. prunioides* (79 mg/g GAE) and *M. comosus* (64.7 mg/g GAE).

**Fig. 1:**
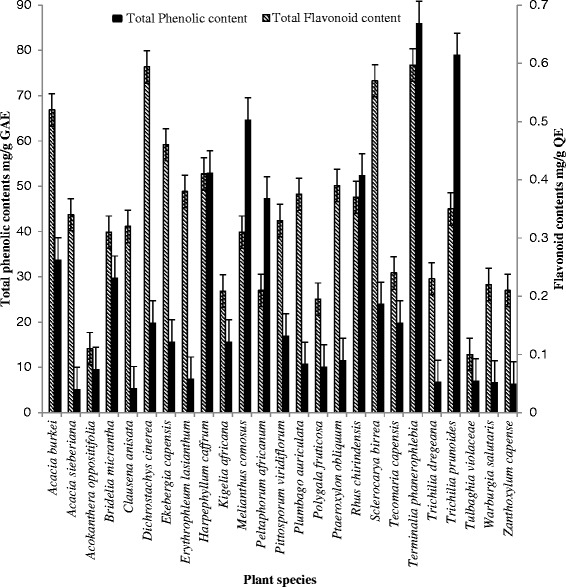
Relationship between total phenolic and flavonoid contents. Results indicated that there was no correlation between the total phenolic content and flavonoid content of the extracts tested (R^2^ = 0.05)

In terms of flavonoid content, the three highest yields were obtained from *D. cinerea* (0.54 mg/g QE), *T. phanerophlebia* (0.53 mg/g QE) and *S. birrea* (0.52 mg/g QE), respectively (Fig. 1). The overall results suggest that generally, there was poor correlation between total phenolics and flavonoid contents in the selected plant species (R^2^ = 0.05); however, *T. phanerophlebia* seems to be an exception. Not much study has been done on phyto-chemical screening of the leaves of *T. phanerophlebia,* but available literature data indicates the presence of triterpenoids and tannins [10]. The dried leaves of the plant are generally used as decoction in water to treat rheumatism, stomach pains and diarrhoea [38]. The high content of total phenolics and flavonoids, possibly tannins, triterpenoids and other secondary metabolites may be responsible for its therapeutic uses.

Data from literature sources on the secondary metabolites present in the leaves of *T. prunioides* is scarce. Its antibacterial [39], Thin Layer Chromatography profile and antifungal activity [40], and antioxidant activity [41] has been reported. However, the dried leaves are used as decoction traditionally for the relief of stomach pains. Our study indicated that it contained relatively high amounts of phenolic compounds, possibly flavonoids, tannins and terpenoids, this may be responsible for the antimicrobial and antioxidant activity. The third plant species with a high phenolic content among the selected plants was *M. comosus.* Potential anti-fungal and lipoxygenase inhibitory properties of this plant species have already been reported. This may be associated with its flavonoid and cardiac glycoside content [42]. Phenolic compounds, especially flavonoids are well known for their anti-oxidant activitiy and lipoxygenase enzyme inhibitory activity [43].

### Anti-inflammatory activities

**Fig. 2:**
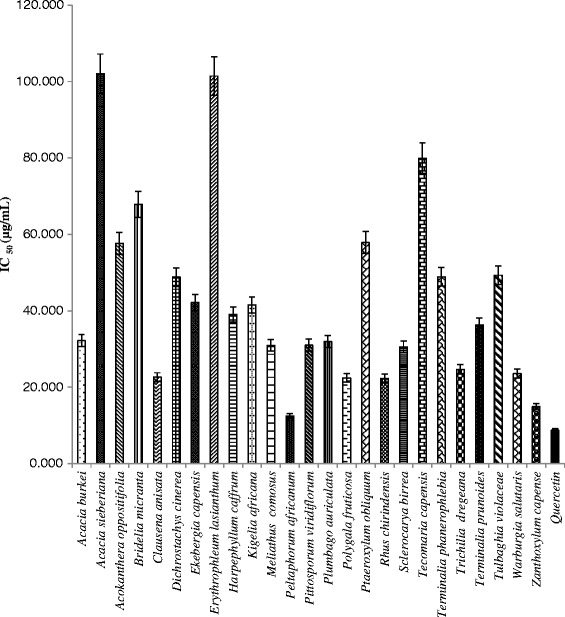
Inhibitory activities of crude plant extracts on 15-LOX. The extract with the highest inhibitory activity on 15-LOX was obtained from *P. africanum* (IC_50_ = 12.42 μg/mL) compared with quercetin controls (IC_50_ = 8.75 μg/mL)

The main objective of the study was to evaluate the anti-inflammatory activity of the selected extracts using the anti-15 LOX model of inhibition. Therefore the three plants extracts with promising inhibitory activity of 15-LOX were selected for further investigation. As illustrated in Fig. 2, crude extracts harvested from two of the plant species tested, *P. africanum* (IC_50_ = 12.42 μg/mL) and *Z. capense* (IC_50_ = 14.92 μg/mL), had promising 15-LOX inhibitory activities compared with quercetin (IC_50_ = 8.75 μg/mL) used as a positive control. These complex crude extracts may contain compounds with higher activity than quercetin. These results suggest that the bioactive constituent(s) of *P. africanum* had both antioxidant and anti-inflammatory activities. Antioxidants act by scavenging free radicals such as reactive oxygen species, hydroxyl radicals and nitric oxide while anti-inflammatory mediators act by modulating the activities of pro-inflammatory enzymes and cytokines.

The lipoxygenase group of enzymes (5, 8, 12 and 15-LOX) plays a role in many inflammatory disorders. The isomeric enzyme, 15-LOX is an important enzyme involved in the synthesis of leukotrienes from arachidonic acids. Biologically active leukotrienes are mediators of many pro-inflammatory and allergic reactions, therefore the inhibition of the synthesis of leukotrienes by 15-LOX is considered as one of the therapeutic strategies in the management of inflammatory conditions [17, 24]. Assessment of extracts derived from more than 180 different plant species indicated their potential dual COX/LOX inhibitory capacity [24]. Extracts or compounds from plants inhibiting the pro-inflammatory activities of these enzymes may contain potential leads or templates for the development of potent anti-inflammatory drugs [44]. Further work is required to properly characterize the compound(s) responsible for the anti-inflammatory principles in these plant species, and also understand their mechanisms of action. The three plants extracts with promising inhibitory activity on 15-LOX were selected for further investigation.

### Inhibition of nitric oxide (NO) production by the three extracts with promising 15-LOX inhibitory activity

The inhibitory activity of the extracts on NO production by induced RAW 264.7 macrophage cell lines is presented in Table 2. *Clausena anisata* crude acetone extracts had the best inhibitory activity on NO production (96.9 % inhibition/ 71.3 % cell viability) at 6.25 μg/mL compared with *P. africanum* (91.3 %/ 65.7 %), *Z. capense* (62.2 %/ 82.5 %) and quercetin (91.1 %/ 73.8 %) respectively (Table 2). Extracts with good inhibitory activity on NO production and a low cytotoxicity are more useful. Release of NO promotes inflammation, therefore extracts that could act as scavengers of NO, or inhibitors of its production, especially with corresponding low cytoxicity could be used to mitigate the propagation of inflammation by NO. The inhibition of NO production by extracts derived from medicinal plants may be due to the inhibition of inducible nitric oxide synthase activity and/or its expression [30, 31].

**Table 2:** Inhibitory activities of *Peltophorum africanum (PA),* *Clausena anisata (CA) and* *Zanthoxylum capense* (ZC) on the LPS-activated NO production in RAW 264.7 macrophages

Samples	Concentration (μg/mL)	NO (μM)	% NO inhibition	% Cell viability
*Peltophorum africanum*	25	0.60 ± 0.02	92.32	66.64
	12.5	0.39 ± 0.14	94.92	79.71
	6.25	0.67 ± 0.42	91.33	65.71
	3.12	1.97 ± 0.24	74.62	60.93
*Clausena anisata*	25	0.94 ± 0.07	87.86	3.43
	12.5	0.38 ± 0.25	95.17	63.31
	6.25	0.24 ± 0.14	96.90	71.29
	3.12	0.55 ± 0.08	92.94	80.31
*Zanthoxylum capense*	25	1.06 ± 0.58	86.38	72.79
	12.5	1.31 ± 0.51	83.16	90.14
	6.25	2.94 ± 0.46	62.23	82.45
	3.12	5.22 ± 0.67	32.88	79.24
Quercetin	25	0.35 ± 0.10	95.54	49.33
	12.5	0.30 ± 0.08	96.16	60.69
	6.25	0.69 ± 0.05	91.08	73.76
	3.12	2.50 ± 0.48	67.93	73.10

Values are expressed as mean ± SD

### Antioxidant activities

Phytochemical evaluation of extracts derived from *P. africanum* has yielded bergenin [45] and betulinic acid [46]. Secondary metabolites are stored in various parts of the plant; however coumarins constitute the major compounds in the leaves [47]. The plant is widely used traditionally for treating wounds, back and joint pains and dysentery, among others [48], but reported biological activities of the extracts are limited to antimicrobial activity [49]. The bioactive compounds responsible for the observed effects have not been properly characterized and the mechanism of activity has not been explored. In the case of *Z. capense,* biological activities such as anti-mycobacterial [50] and anti-proliferative effects [51] have been reported, subsequent to a bio-assay guided isolation of six alkaloids from the roots of the plant [52].

Extracts of *P. africanum* had the best antioxidant activity among the three extracts that were tested (Table 3). With IC_50_ of 4.67 ± 0.31 and 7.71 ± 0.36 μg/mL using the DPPH and ABTS assays respectively, the results were comparable to that of Trolox positive controls (2.74 ± 0.08 and 7.21 ± 0.42 μg/mL). This is consistent with the findings of Bizimenyera, 2007 [53]. An extensive review of extracts of African medicinal plants with potent anti-oxidant activities by Atawodi [54] indicated that the mechanism(s) of action extracts was by free radical scavenging. In addition, the synergistic effects of natural products also enhance their antioxidant activities [53]. These results suggest that the bioactive constituent(s) of *P. africanum* had both antioxidant and anti-inflammatory activities. Antioxidants acts by scavenging free radicals such as reactive oxygen species, hydroxyl radicals and nitric oxide while anti-inflammatory mediators act by modulating the activities of pro-inflammatory enzymes and cytokines. Accumulation of free radicals result in cellular injury, and may be the cause of many diseases.

**Table 3:** Cytotoxicity, antioxidant activity, total phenolics and total flavonoids content of acetone extracts of *Clausena anisata, Peltophorum africannum and Zanthoxylum capense*

Samples	VERO IC_50_ (μg/mL)	DPPH IC_50_ (μg/mL)	ABTS IC_50_ (μg/mL)	FRAP IC_50_ (μgFe (II)/g)	TPC (mg GAE/g)	TFC (mg QE/g)
*Clausena anisata*	23.19 ± 0.58	119.36 ± 3.78	64.08 ± 2.61	146.52 ± 11.97	109.63 ± 7.62	159.01 ± 1.88
*Peltophorum africanum*	103.45 ± 0.41	4.67 ± 0.31	7.71 ± 0.36	434.54 ± 29.82	255.26 ± 28.69	80.00 ± 8.06
*Zanthoxylum capense*	>1000	138.78 ± 13.24	132.10 ± 8.10	93.96 ± 7.68	372.27 ± 16.06	38.59 ± 6.65
Trolox		2.74 ± 0.08	7.21 ± 0.42	Nd	Nd	Nd
Ascorbic. Acid		2.92 ± 0.14	13.57 ± 0.44	Nd	Nd	Nd

Values are expressed as mean ± SD

Nd: not determined

### Cytotoxicity

Our results indicated that extracts of *Z. capense* had the lowest cytotoxicity on Vero Monkey kidney cell lines (Table 3) among those tested (IC_50_ > 1000 μg/mL). *Peltophorum africanum* extracts also had a relatively low toxicity of with an IC_50_ of 103 μg/mL that was comparable to values in an earlier report [53]. The safety of herbal remedies remains a concern because few reports exist on the safe use of these products. Many extracts have been shown to contain potentially harmful substances that could impact adversely on human health when consumed [55]. Although, our study suggests that extracts of *Z. capense* had low toxicity on Vero cell lines (≥1000 μg/mL) (Table 3), this observation has not yet been confirmed using in animal studies.

## Conclusions

Our results provide further scientific evidence supporting the use of *P. africanum, Z. capense* and *C. anisata* as anti-inflammatory and pain relief remedies in traditional medicine. To be used as herbal products the safety in animal experiments have to be confirmed. The good inhibitory activity of crude extracts containing many other compounds on 15-LOX inhibition in these plant species means that it probably contains compounds with excellent activities. Further work is required to isolate, identify and characterize the bioactive compounds that are responsible for the activities. Once the active compounds have been isolated the mechanism of activity can be examined.
